# Poster Session I - A43 GUT–BREAST AXIS: DIETARY FLAXSEED MODIFIES MICROBIOTA–MAMMARY MICRORNA NETWORKS LINKED TO BREAST CANCER IN AN INTESTINAL REGION-SPECIFIC MANNER

**DOI:** 10.1093/jcag/gwaf042.043

**Published:** 2026-02-13

**Authors:** Y Chen, E Karanxha, D Wu, L Thompson, E M Comelli

**Affiliations:** University of Toronto Department of Nutritional Sciences, Toronto, ON, Canada; University of Toronto Department of Nutritional Sciences, Toronto, ON, Canada; University of Toronto Department of Nutritional Sciences, Toronto, ON, Canada; University of Toronto Department of Nutritional Sciences, Toronto, ON, Canada; University of Toronto Department of Nutritional Sciences, Toronto, ON, Canada

## Abstract

**Background:**

The beneficial effects of flaxseed (FS)-derived lignans are unlocked by the gut microbiota via FS secoisolariciresinol diglucoside (SDG) metabolism into enterolignans. We found that the cecal microbiota and mammary gland (MG) microRNA (miRNA) signatures are related and modified by FS towards an antioncogenic phenotype in female mice. Relationships in distal gut regions harboring a different microbiome are unknown.

**Aims:**

To identify the relationships between fecal microbiota and MG miRNome in response to FS and SDG, and to compare them with the cecal microbiota–MG miRNome signatures.

**Methods:**

MiRNA (NanoString) and microbiota (16S rRNA sequencing) data previously generated from female C57BL/6 mice receiving a control diet, or 10% dietary FS or equivalent amount of SDG were used. Spearman correlations were assessed by differential gene correlation analysis (R). Predicted target genes of miRNAs in significant correlations were identified with miRDB version 6 (Target Score ≥ 95). Their human orthologues were used for pathway enrichment analysis (PathDIP 5, Bonferroni corrected p-value < 0.05) across 12 databases.

**Results:**

139, 25 and 35 significant fecal taxa-MG miRNA correlations were found in the control, FS and SDG groups, respectively. 106 and 110 correlation changes (inverted, nullified or emerged) were identified in the FS and SDG groups compared to BD, and 48 between SDG and FS. However, no correlation changes were shared between the BD-FS and FS-SDG groups. Interestingly, 32 taxa:miRNA correlations were conserved between cecum and feces in the control group, but none in the FS nor SDG groups. Genes targeted by miRNA in significant fecal correlations were enriched in 393, 117, and 149 pathways in the BD, FS, and SDG groups, respectively. The PI3k-Akt-mTOR and RUNX1 pathways were common to the three diets in both cecum and feces.

**Conclusions:**

Microbiota and MG miRNA relationships vary along the intestinal tract and respond to FS in a distinct manner. As SDG alone did not fully recapitulate FS effects, other FS components are likely contributors, potentially through further metabolism in more distal regions. Multiple intestinal sites may contribute to the gut-breast axis in a microbiota-dependent manner. The overlapping targeting of breast-cancer related pathways by miRNA in different taxa correlations suggests that these effects are prioritized along the large intestine but modifiable by FS and its lignans.

**Funding Agencies:**

NSERC

